# Enhancement of the Corrosion Properties of Al–10%Si–2%Cu Alloys with La Addition

**DOI:** 10.3390/ma17112496

**Published:** 2024-05-22

**Authors:** Kyeonghun Kim, Uro Heo, Haewoong Yang, Namhyun Kang

**Affiliations:** 1 Pohang Institute of Metal Industry Advancement (POMIA), 56, Jigok-ro, Nam-gu, Pohang-si 37666, Republic of Korea; kkh@pomia.or.kr (K.K.); uro@pomia.or.kr (U.H.); hwyang@pomia.or.kr (H.Y.); 2 Department of Materials Science and Engineering, Pusan National University, 2, Busandaehak-ro 63beon-gil, Geumjeong-gu, Busan 46241, Republic of Korea

**Keywords:** aluminum alloy, lanthanum, microstructure, corrosion properties, intermetallic compound

## Abstract

Al–10%Si–2%Cu alloys have been widely used in high-value industries (e.g., aerospace and automobiles) because of their lower specific gravity; however, galvanic corrosion rendered these alloys to have poor corrosion resistance. Therefore, the microstructure and corrosion properties of Al–10%Si–2%Cu alloys were investigated with respect to the lanthanum (La) content. All Al alloy samples were synthesized using gravity casting, with added La contents of 0.00, 0.25, 0.50, 0.75, and 1.00 wt%, and were characterized using microstructural characteristics analysis and electrochemical tests. Adding 0.5 wt% La (xLa-0.5) indicated the finest structure, which had a 4% lower α-Al area fraction than the La-free alloy (xLa-0). However, the area fraction of a 1 wt% La-added (xLa-1) alloy was 2.4% higher than that of xLa-0. The corrosion current density (*I*_corr_) of the xLa-0.5 was 1.09 μA/cm^2^, representing a 68% decrease as compared to that of xLa-0, and xLa-0.5 reached the highest polarization resistance value (7.32 × 10^3^ Ω·cm^2^). The improvement in corrosion resistance of xLa-0.5 was due to the rapid and dense formation of a passivation layer induced by its fine structure, as well as the precipitated phase by enhancing the dispersibility of Cu.

## 1. Introduction

Al–10%Si–2%Cu alloys have applications in high-value industries, such as aerospace and automobiles, because of their lower specific gravity than steel. In addition, these alloys have exceptional productivity, superior mechanical properties, and an aesthetically pleasing surface finish, making them suitable for mass production. Recently, the automobile industry has been focusing on developing eco-friendly cars and is primarily using Al–10%Si–2%Cu alloys [1,2]. However, Al–Si–Cu alloys used in die casting have poor corrosion resistance caused by galvanic corrosion due to potential differences with the α-Al phase, including the Al_2_Cu precipitated phase [3]. These limitations affect their application, especially in parts exposed to corrosive environments.

To address these limitations, M. Matejka’s research group suggested the corrosion rate of Al-Si5Cu2-Mg alloy according to Zr content and showed the corrosion resistance was improved if 0.1 wt% Zr was added [4]. P, Zhou et al. reported that the addition of Cr to Al-Si-Cu-Mg alloy not only improves corrosion resistance but also mechanical properties [5]. In the aluminum alloy, various studies have been reported on enhancing the corrosion resistance and mechanical properties of aluminum (Al) alloys by modifying their microstructure through the addition of rare-earth elements [6,7,8,9,10]. A representative example is a research study on grain deformation through La, Ce, Y, and Nd addition. The corrosion is mainly concentrated at the grain boundary between the intermetallic compound and matrix aluminum [5,9]. In research on the corrosion of aluminum with the addition of rare-earth elements, it is reported that the low potential difference between the new precipitate phase and aluminum shows a lower corrosion rate compared to the alloy without rare-earth elements and improves corrosion resistance properties [11,12,13]. However, most rare-earth elements have high costs and limited reserves, making their application to Al alloys challenging. Recently, many studies have investigated the improvement of the Al alloy corrosion resistance and mechanical properties using La, which has the largest reserves among rare-earth elements [14].

When La is added to a 6xxx Al alloy, the castability is improved, and the precipitation of α-AlFeSi phases can be promoted while suppressing that of harmful lath-like β phases [15,16,17]. In addition, when La is added to an Al–Cu alloy, the dislocation and grain boundary movement can be effectively suppressed. Furthermore, a potential polarization test and impedance analysis showed improved corrosion properties [18]. In this study, the corrosion properties of La-added Al-Si-Cu alloy were investigated through microstructural characteristics analysis and electrochemical tests.

## 2. Experimental Section

Al–10%Si–2%Cu alloys as master alloys were investigated, and all ingots with the Al–10%Si–2%Cu–xLa composition were synthesized using Al–20%La and Al–10%Sr as master alloys. A specific amount of Al–10%Sr was added to all samples to achieve a grain refinement effect. The Al alloy was produced as a rectangular column (1200 mm × 600 mm × 300 mm) using gravity casting. During this process, the molten metal temperature was maintained at 780 °C, the mold temperature was set to 200 °C, and air cooling was applied. The added La amounts were 0.00, 0.25, 0.50, 0.75, and 1.00 wt%, respectively, denoted as xLa-0, xLa-0.25, xLa-0.5, xLa-0.75, and xLa-1. Table 1 summarizes the ingot compositions measured using wavelength dispersive X-ray fluorescence (WDXRF; M4 Tornado, Bruker, Berlin, Germany). 

The specimens mounted for microstructure analysis were made into single specimens according to the La content. All test specimens were obtained by processing the central section to eliminate impurity influences for a characteristic evaluation sample. Polishing was conducted with 300–4000 grit SiC abrasive paper, followed by the final step with oxide polishing suspension (OPS). The microstructure was examined using a light optical microscope (OM), and the α-Al fraction was analyzed in five random areas using an image analysis program (ImageJ; version 1.35j). The precipitated phase was analyzed using a scanning electron microscope (SEM, SU5000, Hitachi, Tokyo, Japan) equipped with energy-dispersive X-ray spectroscopy (EDS) and electron backscattered diffraction (EBSD). An X-ray diffractometer (XRD, JP/Max-3A, Rigaku, Tokyo, Japan) was used to verify the phase analysis. Differential scanning calorimetry (DSC) experiments were conducted using a computerized differential scanning calorimeter (DSC 404 F1, Pegasus^®^, NETZCHS, Paju, Republic of Korea), and a protective atmosphere of argon (99.9%) with a gas flow of 240.3 mL/min was used. The sample, which was extracted from each master alloy, was analyzed over a temperature range of 60–700 °C at a constant heating and cooling rate of 10 °C/min. The samples with xLa alloys were each subjected to three experiments. An electrochemical experiment (Autolab/PGSTAT302N, Metrohm, Herisau, Switzerland) was performed using a three-electrode configuration cell to evaluate the corrosion properties. The electrode exposure area was 50 mm^2^, and the counter and reference electrodes were a Pt plate and an Ag/AgCl electrode, respectively. Potentiodynamic polarization scans were carried out with a scan rate of 0.17 mV/s in the range from −1.5 V to 0 V. The temperature of bath and the electrolyte used were 25 °C and 0.05 M NaCl, respectively. Additionally, the experiment was performed by exposing a half of the specimen (100 mm^2^) to the electrolyte. Before the corrosion experiment, the solution was purged with nitrogen gas for 15 min to remove dissolved oxygen. After performing the corrosion test, Autolab NOVA 2.1 was used to interpret the results. Additionally, XRD, DSC, and electrochemical polarization tests were performed once, three times, and twice, respectively.

## 3. Results and Discussion

**Figure 1:**
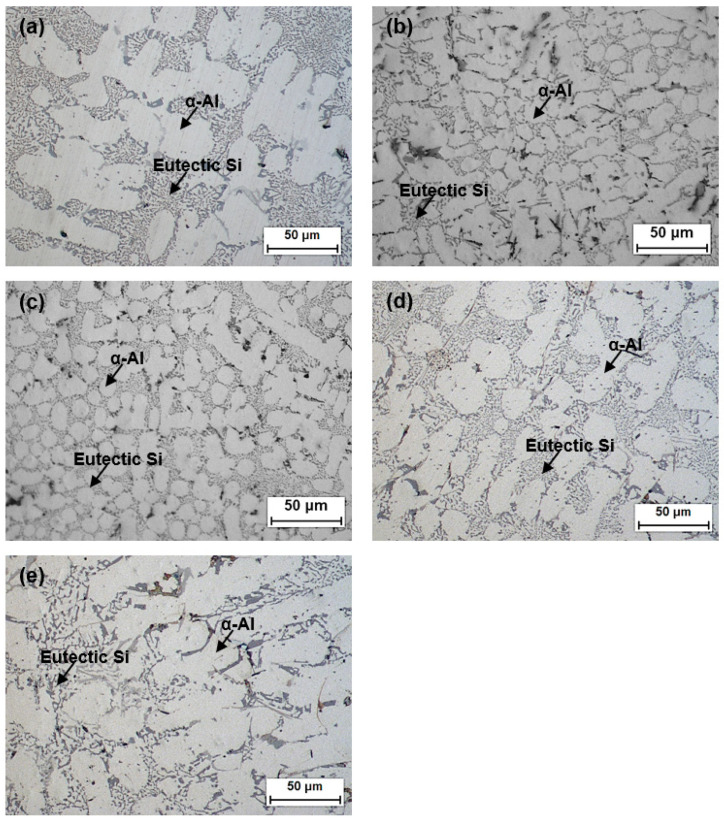
Optical microscopy analysis results for xLa alloys: (**a**) xLa-0, (**b**) xLa-0.25, (**c**) xLa-0.5, (**d**) xLa-0.75, and (**e**) xLa-1.

Figure 1 shows the microstructure of the xLa alloys (*x* = 0.00, 0.25, 0.50, 0.75, 1.00). For the Al–Si–Cu alloy without La (xLa-0), a clearly connected dendritic form, which is typical of dendritic casting structures, was observed, as shown in Figure 1a. In contrast, the alloys with 0.25 and 0.50 wt% La (xLa-0.25 and xLa-0.5, respectively) exhibited a mixture of dendritic and equiaxed structures in the α-Al phase, as shown in Figure 1b and Figure 1c, respectively. The size of the equiaxed structure increased in xLa-0.75 (Figure 1d). The dendritic structure was dominant in 1.00 wt% La (xLa-1) in Figure 1e, similar to that in xLa-0 (Figure 1a), and eutectic Si was observed in coral-like shapes. The needle-like phase is primarily observed in eutectic Si structures in Al–Si alloys, and it was observed in the xLa-1 alloy. This is assumed to be influenced by Sr, which contributes to the grain refinement [19]. In addition to the effect of Sr addition, different grain refinements were observed depending on the presence and wt% of the added La.

**Figure 2:**
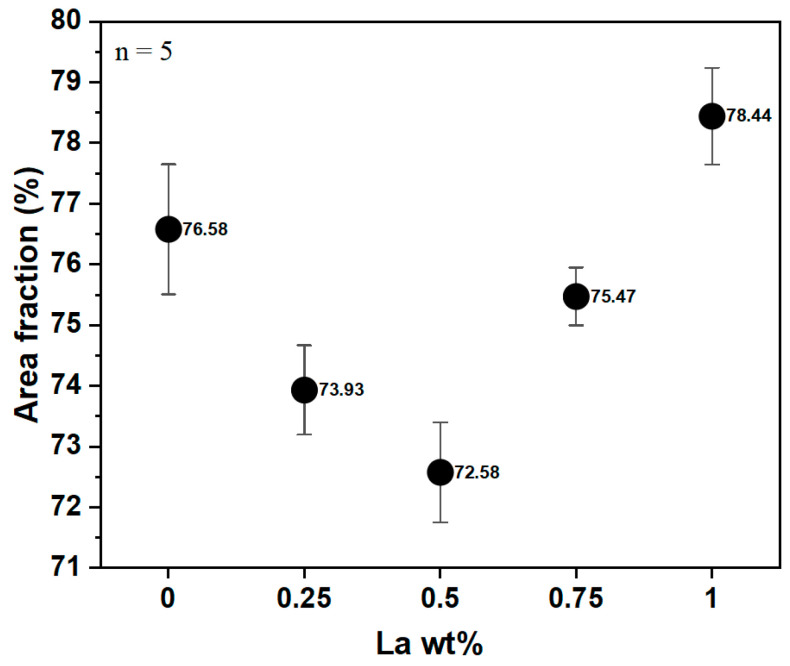
α-Al fraction as a function of La addition.

The grain refinement on α-Al was quantitatively analyzed using the Otsu threshold, and the results are shown in Figure 2. Table 2 summarizes the minimum, average, maximum and dispersion values of the area fraction of α-Al according to La content. The grain refinement of xLa-0.5 was observed, resulting in a 4% lower α-Al area fraction than that of the xLa-0 alloy. In addition, the structure coarsening of xLa-1 was also observed, with a 2.4% higher fraction than that of xLa-0. The precipitated phase produced through La addition was directly related to the grain refinement of the Al alloy. Generally, it is difficult to promote nucleation due to the continuous crystal growth, and there was an optimized La content amount to induce grain refinement and coarsening [20].

La atoms easily aggregate at the solid–liquid interface when Al solidifies, causing compositional undercooling due to the difference between the thermal equilibrium state temperature and the actual cooling temperature [21,22]. This compositional supercooling effect promotes the formation of the La precipitate phase, suppresses the formation of α-Al, and achieves the grain refinement effect. The growth restricting factor (GRF) represents the influence of solutes on grain refinement, and its formula is shown in Equation (1), where mi, C0, and ki are the liquidus slope, initial solute concentration, and partition coefficient, respectively. In Al–La alloy, the mLa and kLa values are known to be −2.034 and 0.003, respectively, and the GRF of 0.5 wt% La added was calculated to be 2.028, which was 0.186 higher than that of the La-free alloy [23,24]. If the GRF is high, the liquid metal around the nucleation spot is cooled more quickly due to the rapid formation of compositional supercooling, and thus, stable nucleation is possible.

(1) $$GRF=\sum_{i}m_{i}C_{0}(k_{i}-1)$$

The contact angle (*θ*) as represented in Equation (2) is directly associated with undercooling according to the heterogeneous nucleation theory, in which $T_{N}^{\alpha -Al}$ and $T_{L}^{\alpha -Al}$ are the nucleation temperature of α-Al and the liquidus temperature of the xLa alloy, respectively.

(2) $$f\left(\theta \right)=\frac{2-cos\theta +{cos\theta }^{3}}{4}=27({{∆T}^{\alpha -Al})}^{2}T_{N}^{\alpha -Al}/4({T_{L}^{\alpha -Al})}^{3}$$

**Figure 3:**
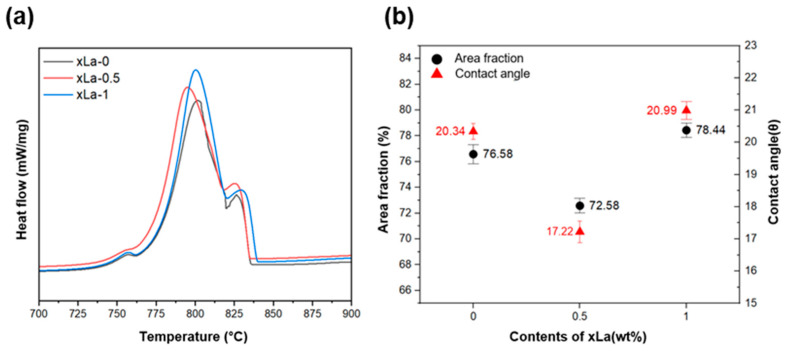
(**a**) Differential scanning calorimetry analysis results for the La contents, and (**b**) a comparison of the contact angle (*θ*) and area fraction.

The average contact angle was calculated from the DSC experiments, as shown in Figure 3 and summarized in Table 3. Through the contact angle, the addition of La causes lower supercooling during nucleation, resulting in the low area fraction [25,26,27]. The calculated average contact angle values were analyzed to be 20.3, 17.2, and 20.9 for La contents of 0, 0.5, and 1 wt%, respectively, and the lowest angle was observed at 0.5 wt%. It was observed that the α-Al growth was suppressed at xLa-0.5 due to the supercooling effect, resulting in the formation of a fine structure. In a previous study [28], the contact angle of xLa-0 and xLa-0.5 decreased to 13.5° and 6.3°, respectively. It is verified that adding La reduced the driving force required for nucleation by reducing the contact angle during the formation of heterogeneous nuclei, thereby facilitating the formation of α-Al and influencing the grain morphology. However, if the La content is over 0.50 wt%, the supercooling effect diminishes, leading to a coarsening phenomenon. Therefore, the contact angle showed the same trend with the area fraction of α-Al.

**Figure 4:**
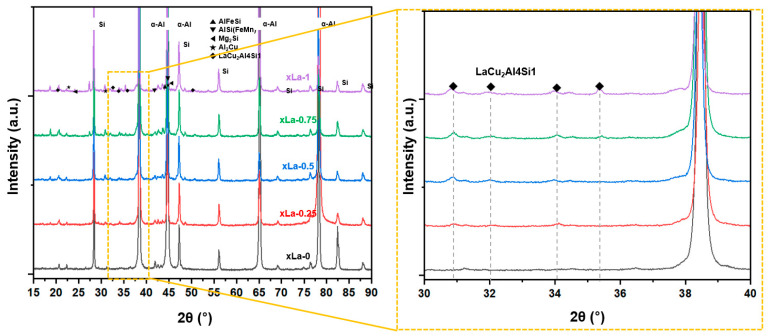
X-ray diffraction analysis for the Al–Si–Cu alloy with various La addition amounts.

Figure 4 shows the phase analysis using XRD. Precipitated phases mainly found in Al–Si–Cu alloys were observed, such as Al_2_Cu, AlSi(FeMn), and Mg_2_Si. In addition, the LaCu_2_Al_4_Si phase was also observed in the La-added alloy (xLa-0.25–1.0). The peaks associated with the LaCu*_2_*Al_4_Si phase in the 30°–35° range exhibited variations depending on the La content.

**Figure 5:**
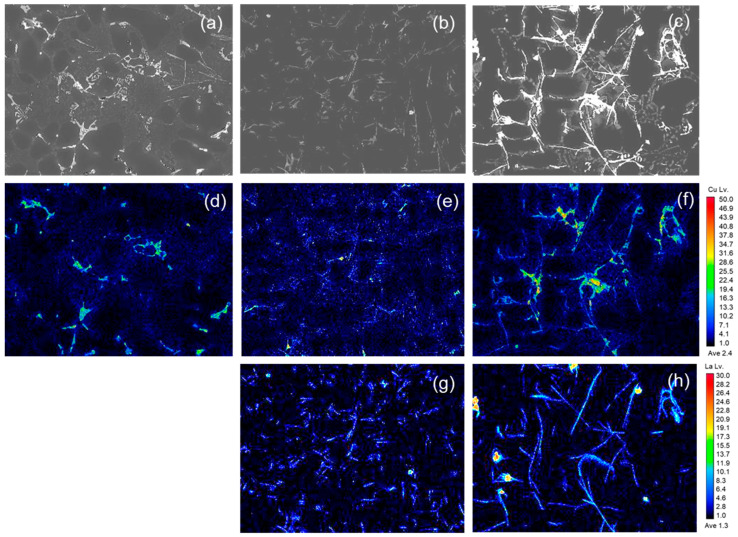
The electron probe microanalyzer (EPMA) mapping analysis of Cu with varying La contents: (**a**–**c**) SEM images of xLa (0, 0.5, and 1.0 wt%, respectively); (**d**–**f**) EDS mapping images of Cu (0, 0.5, and 1.0 wt%, respectively); and (**g**,**h**) xLa (0.5 and 1.0 wt%, respectively).

The microstructure and Cu mapping images are shown in Figure 5 to gain a more detailed understanding of the phase behavior of the La-added Al–Si–Cu alloys. Upon adding La and Cu, a precipitated phase was primarily formed at the grain boundaries, and an increase in the added La content resulted in the agglomeration of the La and Cu precipitated phase. The atomic size of La (0.188 nm) has a significant difference of ~60% as compared to that of Al (0.118 nm), which means that, due to the difference in atomic radii, most of the added La precipitates in the Al matrix without forming a solid solution. In addition, the mixing enthalpy of La–Cu (−21 kJ/mol) is lower than that of Al–Cu (−1 kJ/mol) [29]. Therefore, La can easily dissolve in the Al matrix.

**Figure 6:**
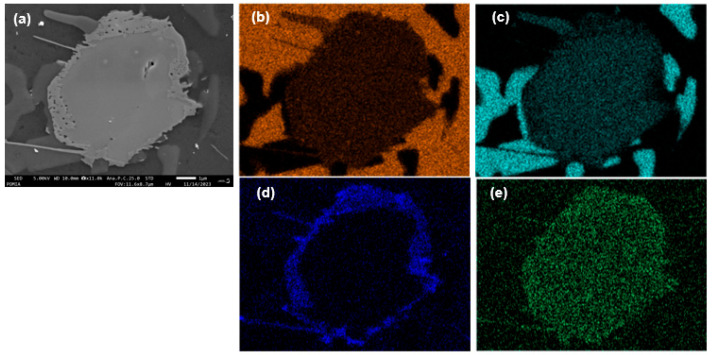
Back scattered SEM images aluminum alloy. (**a**) xLa-0.6, Energy-dispersive X-ray spectroscopy (EDS) analysis of LaCu_2_Al_4_Si, (**b**) Al, (**c**) Si, (**d**) Cu, (**e**) La.

The observed La phase was confirmed as LaCu_2_Al_4_Si, as shown in Figure 6. Figure 6a shows the LaCu_2_Al_4_Si phase by SEM, and Figure 6b shows the results of EDS analysis of the LaCu_2_Al_4_Si phase. It reveals a core–shell structure with observed La and Cu in each area of the core and shell. The precipitated phase mentioned above has rarely been reported in previous studies, and in existing research on Al–Mg–Si alloys with La addition, the content of Cu is typically low, ranging from 0.10% to 0.40%. Therefore, the variation in Cu due to La addition has not been addressed as a significant issue. However, the Al-Si alloy used in this study commonly contains about 2.0 wt% Cu, deviating from the content reported in previous studies in the literature [14,30]. This discrepancy in the Cu content was observed as a distinctive trend in this study. A coarse phase was observed in the alloy without La addition (Figure 6a); however, the refinement was observed at 0.5 wt% La in the Cu phase. In addition, a significant amount of Cu phase similar to that of the xLa-0 alloy was analyzed in the xLa-1 alloy, which is presumed to be influenced by the core–shell structure of the LaCu_2_Al_4_Si phase formed due to La addition.

### Corrosion of Al Alloy with La Addition

**Figure 7:**
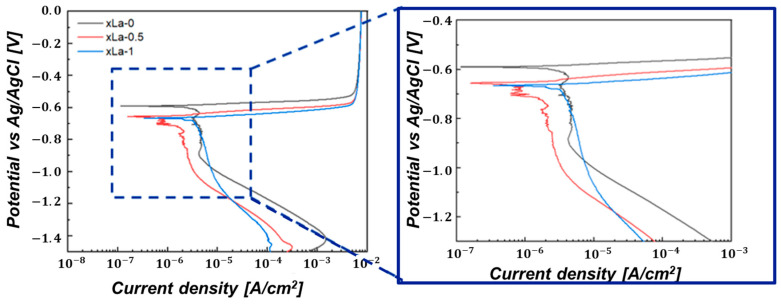
Polarization curves of the Al–Si–Cu–xLa alloys in 3.5 wt% NaCl solution.

The corrosion properties of xLa alloy were evaluated through electrochemical polarization tests. In the Tafel polarization curve, as shown in Figure 7, the anode part shows closely similar behavior, and when La is added, the cathode part exhibits a shift to the left, indicating that the corrosion rate decreases. Table 4 indicates the results of determining the corrosion resistance for each alloy according to the presence and absence of La addition and amount in Al–Si–Cu alloy. Generally, it is known that the corrosion resistance is excellent whether the corrosion potential (*E*_corr_) is high or the corrosion current density (*I*_corr_) is low. It could be observed that there was little difference in *E*_corr_ within the standard error range through the corrosion potential and corrosion current density, as shown in Table 4. As a result of analyzing the corrosion current density tendency with respect to the La amount, the corrosion current density was 3.32 μA/cm^2^ at 1 wt%, showing a corrosion current density closely similar to that at 0 wt%. The corrosion current density in the xLa-0.5 alloy was the lowest at 1.09 μA/cm^2^, decreasing by approximately 68% compared to the xLa-0 alloy.

To analyze resistance characteristics based on the presence of the added La and its amount, the polarization resistance was calculated using the Stern–Geary equation (Equation (3)). In Equation (3), *I*_corr_ is the corrosion current density (A/cm^2^), and *β*_a_ and *β*_c_ are the Tafel slopes of the anode and cathode, respectively [31]. In general, it is known that polarization resistance and corrosion rate are inversely proportional; the results of calculating the polarization resistance are shown in Table 4. The polarization resistance of 0 wt% and 0.5 wt% La is 7.32 × 10^3^ Ω·cm^2^ and 1.97 × 10^3^ Ω·cm^2^, respectively. The tendency of polarization resistance in terms of the La amount showed that the corrosion rate increased as the La amount increased; it looks similar to the corrosion current density trend.

(3) $$R_{p}=\frac{\beta_{a}\times \beta_{c}}{2.3\times I_{corr}(\beta_{a}+\beta_{c})}$$

Furthermore, the corrosion rate (mmy), the annual mass loss rate, was calculated through actual polarization experiments using Equation (4) [32]. In Equation (4), *K* is a constant (3272 mm A/cm^2^ year^−1^) according to the ASTM G 102 standard [33], EW is the equivalent weight (g/eq), *ρ* is the specimen density (g/cm^3^), and *A* is the surface area (cm^2^). As a result, when 0.5 wt% La was added, the annual mass loss rate was the lowest (6.59 × 10^−3^) for each alloy. Although the corrosion rate showed a relatively low value, it decreased by about 68% compared to the alloy without La. Thus, it shows excellent corrosion properties with the lowest annual mass loss rate when 0.5 wt% La is added.

(4) $$Corrosion\;rate=\frac{I_{corr}\times K\times EW}{\rho \times A}$$

**Figure 8:**
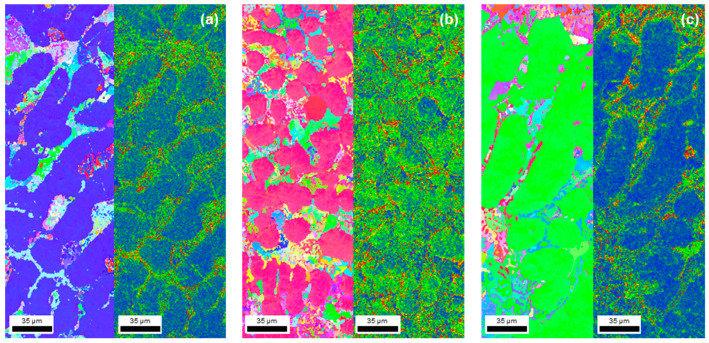
Electron backscattered diffraction (EBSD) analysis results for Al–Si–Cu–xLa alloys in 3.5 wt% NaCl solution: (**a**) xLa-0, (**b**) xLa-0.5, and (**c**) xLa-1 wt% (scale bar: 35 μm).

There have been various studies on the relationship between corrosion rates and changes in the microstructure size of metal materials, but clear conclusions have not yet emerged [34]. However, research on evaluating the corrosion properties of Al alloys with added rare-earth elements like La has reported that corrosion resistance improves as the crystal size decreases. However, from a relatively microscopic perspective, most studies reported that as elements were added, a new precipitated phase was formed, and corrosion was improved because of the effect of the potential difference or the protective film, such as the oxidation layer [34,35]. Figure 8 shows the results of conducting the EBSD analysis to understand the cause of the improvement in corrosion properties from a macroscopic perspective. The trend of dislocation density was observed to be similar to that of the microstructure change. Comparing the dislocation density depending on the presence or absence of La, it was found that the dislocation density of the grains was low at 0 wt% La, whereas, when 0.5 wt% La was added, it increased, along with grain boundaries. In addition, when 1 wt% La was added, the dislocation density decreased again compared to 0.5 wt% La. According to previous research, it is known that corrosion characteristics are improved by the dense shape of the passivation layer depending on the grain refinement [36]. The ionic and electronic conduction influence the corrosion process, and corrosion systems that rely on electronic conduction generally have faster corrosion rates. The finer the grain size, the more dependent on ion conduction [37]. This tendency suggests that although the oxidation is not clearly formed at 0.5 wt% La, the effects of corrosion inhibition and oxidation are not invalidated, and corrosion can be effectively inhibited [38,39]. As a result, grain boundaries are increased, and repassivation occurs at a relatively high rate, improving the corrosion properties because the grain size is decreased through the corrosion resistance from the polarization curve. As shown in Table 4, the corrosion resistance value of 0.5 wt% La was 26.9% higher than that of the alloy without La.

**Figure 9:**
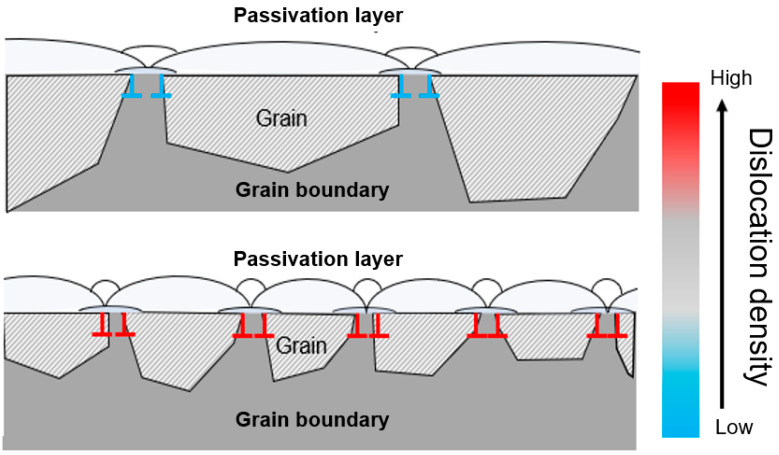
Schematic diagram of passivation layer formation according to the grain size.

Figure 9 shows a schematic diagram of the principle of passivation layer formation according to the dislocation that changes due to the grain size. The passivation layer is formed through diffusion, and it is known that faster and more dense passivation formation is possible in a fine-grain structure, as shown in Figure 9 [40,41,42,43]. Additionally, the flattening of the anode curve in Figure 7 suggests that corrosion can be effectively reduced without showing the oxidation in the electrochemical test. Therefore, this study suggests that when 0.5 wt% La was added, a dense oxide film was quickly formed due to the grain refinement effect, thereby improving the corrosion resistance. In addition, the standard electrode potential of Cu is known to be +337 mV, which is identified as the primary cause of corrosion when added to Al alloys (−166 mV). This study analyzed a lower corrosion potential of up to 0.5 wt% due to the Cu dispersion effects resulting from the LaCu_2_Al_4_Si formation. However, when more than 0.5 wt% La was added, the corrosion properties were not improved because of the coarsening of the precipitated phase and the agglomeration of Cu.

## 4. Conclusions

This study compared the corrosion properties of Al alloy as a function of La contents and derived the optimal La amount in the Al alloys. As La was added up to 0.5 wt%, the α-Al area fraction was 4% lower than that of the alloy without La (xLa-0), resulting in the finest structure. However, the area fraction of the α-Al at xLa-1 was 2.4% higher than that at xLa-0, indicating a coarsening phenomenon. This effect was attributed to compositional supercooling, and the supercooling effect led to rapid growth and the formation of a fine structure up to 0.5 wt% La, while additional La resulted in a decrease in supercooling and a coarser structure. With xLa-0.5, the corrosion current density decreased by ~68% compared to xLa-0, and the polarization resistance reached its highest value at 7.32 × 10^3^ Ω·cm^2^. The improved corrosion properties were attributed to the finest α-Al grains observed at xLa-0.5. This finer structure allowed for the quick and dense passivation layer formation compared to xLa-0, contributing to enhanced corrosion resistance. Additionally, the formation of LaCu_2_Al_4_Si due to La addition was identified as improving the dispersibility of Cu, which is a key element, through the potential difference, further contributing to the enhanced corrosion resistance.

## Figures and Tables

**Table 1:** Chemical compositions of xLa alloys.

Compositions (wt% La)	Label	Si	Mg	Ti	Mn	Fe	Cu	Zn	Sr	La	Al
0	xLa-0	9.91	0.02	0.17	0.70	1.89	1.02	0.02	0.24	0.00	Bal.
0.25	xLa-0.25	9.97	0.02	0.19	0.70	1.86	0.85	0.02	0.22	0.15	
0.5	xLa-0.5	9.79	0.02	0.18	0.70	1.76	0.83	0.02	0.21	0.36	
0.75	xLa-0.75	9.68	0.02	0.19	0.70	1.81	0.80	0.02	0.21	0.61	
1.0	xLa-1	9.56	0.02	0.18	0.70	1.78	0.80	0.02	0.20	0.87	

**Table 2:** α-Al fraction results for various La contents.

Alloys	Min.	Mean.	Max.	Standard Deviation
xLa-0	75.21	76.58	77.85	1.01
xLa-0.25	72.68	73.93	74.56	0.48
xLa-0.5	71.35	72.58	73.50	0.60
xLa-0.75	74.85	75.47	75.98	0.20
xLa-1	77.48	78.44	79.59	0.56

**Table 3:** Contact angle calculated from the differential scanning calorimetry results for various La contents.

Alloys	Max.	Mean.	Min.	Standard Deviation
xLa-0	20.5	20.3	20.2	0.1
xLa-0.5	17.4	17.2	17.0	0.2
xLa-1	21.2	21.0	20.9	0.2

**Table 4:** Simulated parameters of the Tafel polarization curve data obtained for Al–Si–Cu–xLa alloy (wt%) samples in 3.5 wt% NaCl solution.

Alloys	*I*_corr_ (μA/cm^−2^)	*E*_corr_ (V)	*β*_a_ (V/decade)	*β*_c_ (V/decade)	*R*_p_ (Ω·cm^2^)	Corrosion Rate (mmy)
xLa-0	3.43	−0.59	0.017	−0.182	1.97 × 10^3^	2.07 × 10^−2^
xLa-0.5	1.09	−0.65	0.020	−0.239	7.32 × 10^3^	6.59 × 10^−3^
xLa-1	3.32	−0.66	0.024	−0.419	2.97 × 10^3^	2.05 × 10^−2^

## Data Availability

Data are contained within the article.
